# Ultrasound-assisted extraction of alantolactone and isoalantolactone from *Inula helenium* roots

**DOI:** 10.4103/0973-1296.66942

**Published:** 2010

**Authors:** Antoaneta Trendafilova, Christo Chanev, Milka Todorova

**Affiliations:** *Institute of Organic Chemistry with Centre of Phytochemistry, Bulgarian Academy of Sciences, 1113 Sofia, Bulgaria*; 1 *Department of Chemistry, Sofia University “St. Kl. Ohridski”, 1164 Sofia, Bulgaria*

**Keywords:** Alantolactone, gas chromatography, *Inula helenium* L, isoalantolactone, sesquiterpene lactones, ultrasound-assisted extraction

## Abstract

This work deals with ultrasound-assisted extraction (UAE) of alantolactone and isoalantolactone from the roots of *Inula helenium* L., a well-known medicinal plant. The effects of ethanol concentration, extraction time, temperature and number of extraction steps on the extraction yields of both sesquiterpene lactones were investigated. Gas chromatographic (GC) method was used for simultaneous determination of their contents in the corresponding extracts. A comparison with classical extraction methods [maceration, infusion and micro steam distillation-extraction (MSDE)] showed that the amounts of alantolactone and isoalantolactone achieved by UAE with 70 and 96% EtOH for 30 min at room temperature were higher or almost equal to those obtained by maceration for 24 hours.

## INTRODUCTION

Elecampane (*Inula helenium* L.) is a medicinal plant officially listed in some European pharmacopoeias (e.g, PF X, Ned 5, HAB 34).[1] It has been demonstrated that the essential oil and extracts from *Inula helenium* L. roots are rich in eudesmane-type sesquiterpene lactones, mainly alantolactone (1) and isoalantolcatone (2). This pair of structural isomers has been shown to display various pharmacologic activities such as hepatoprotective, antiimflammatory, antitumour, antibacterial, antidematophytic, antifungal activities, etc.[1–5]

Nowadays, renewed interest has grown in the use of medicinal plants as a source of naturally bioactive compounds. Extraction is the first key step in isolation of biologically active compounds. Ultrasound-assisted extraction (UAE) has attracted more and more attention due to its higher extraction efficiency with shorter extraction time compared to traditional methods, such as maceration, soxhlet extraction, etc. It uses sound waves at frequencies above the range audible to humans to disrupt the plant cell wall, thereby enhancing solvent penetration into the plant material and facilitating the release of extracts.[6] In recent years, the application of UAE for the isolation of various biologically active compounds from plant materials has been reported.[7–9]

**Figure 1:**
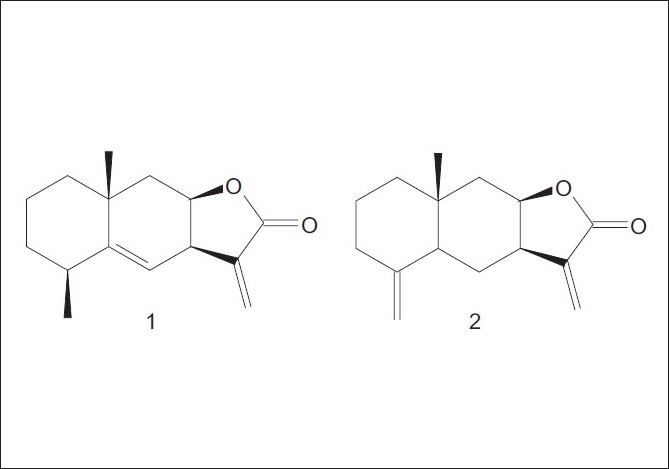
Structures of 1 and 2

In this article, we report a study on UAE and its comparison with classical methods [micro steam distillation-extraction (MSDE), maceration and infusion] for extraction of sesquiterpene lactones 1 and 2 [Figure 1] from *I. helenium* roots. Gas chromatographic (GC) method was used for determination the contents of 1 and 2 in the investigated extracts.

## MATERIALS AND METHODS

### Plant material and reagents

Dried roots of elecampane, *I. helenium* L. were purchased from the herbal drugstore. The plant material was ground and kept in a dark place. Alantolactone and isolantolactone were isolated by the authors and characterized by spectral methods [(infrared (IR), nuclear magnetic resonance (NMR) and mass spectrometry (MS)]. The purities were above 98% as judged by GC analysis. All the solvents used in the experiments were commercially available materials of analytical grade.

### Extraction methods

#### Ultrasound-assisted extraction

UAE experiments were carried out in an ultrasonic bath (UST5.7-150, SIEL, Gabrovo Bulgaria). Plant material (0.5 g) was extracted with 70% aq. EtOH (10 ml) in solid/solvent ratio of 1:20 in an Erlenmayer flask and put in ultrasonic bath for 15, 30, 45 and 60 min. The temperature was kept constant (25 ± 1°C) by periodical adding of ice in the bath. Further, the extract was filtered and solvent was removed under vacuum. Concentrated extract was dissolved in water (10 ml) and extracted with Et_2_O (3 × 10 ml). The obtained extracts were combined, dried (anhydrous Na_2_SO_4_), filtered and concentrated under vacuum. The obtained crude lactone fraction (CLF) was dissolved in MeOH (5 ml) and stored at 4°C prior to GC analysis. The UAE experiments were also performed with 70% aq. EtOH (10 ml) for 30 min at 25, 45 and 65°C, with different volumes of 70% aq. EtOH [solid/solvent ratio 1:10, 1:20, 1:50 and 1:75 (w/v) for 30 min at 25°C] as well as with 20, 40, 70 and 96% aq. EtOH (10 ml) for 30 min at 25 ± 1°C. Three-step UAE was also carried out with 70% aq. EtOH (10 ml) for 30 min at 25 ± 1°C.

#### Maceration

Maceration was performed at room temperature with 70% aq. EtOH. Plant material (0.5 g) was extracted with the corresponding solvent (10 ml, ratio 1:20) for 24 hours. The extracts were worked up as described in for UAE.

#### Micro steam distillation-extraction

Air-dried and ground roots (4 g) were subjected to a MSDE in Likens-Nickerson apparatus for 4 hours using diethyl ether as a solvent. After removing the solvent under N_2_ flow, the essential oil was dried over Na_2_SO_4_ and stored at 4°C until the analysis was carried out. The sample yielded 40 mg (1% w/w) crystalline oil. The obtained oil was further dissolved in MeOH for GC and GC/MS analyses.

#### Infusion

For the preparation of aqueous tea infusion, 1 g of air-dried plant material was infused into 50 ml of boiling distilled water for 10 min, filtered through Whatman no. 4 paper and then concentrated under vacuum to dryness. The obtained residue was worked up as described for UAE.

### Gas chromatography analysis

GC analysis was carried out on HP 5890 A gas chromatograph with flame ionization detector (FID), HP 5 MS capillary column (30 m × 0.25 mm, 0.25 μm film thickness), carrier gas – nitrogen, linear velocity 25 cm/s, split ratio 1:100. Column temperature was programmed from 80 to 210°C at a rate of 15°C/min, to 250°C at a rate of 5°C/min, to 280°C at a rate of 15°C/min and for 9 min at 280°C. Quantitative data were obtained from the electronic integration of the FID peak areas.

#### Quantitative determination of alantolactone and isoalantolactone

Quantitative analysis was carried out using an external standard method. Working solutions containing 4.82, 2.41, 1.20, 0.60, 0.30 and 0.15 mg/ml of 1 and 4.98, 2.49, 1.24, 0.62, 0.31 and 0.16 mg/ml of 2 were prepared from stock solutions in MeOH. Samples of each concentration were injected in triplicate (injection volume 1 µl) and FID peak areas at retention times (RT) of 1 and 2 14.31 and 14.84 min, respectively were used to establish the calibration curves. Linear regression equations were *y* = 80511.577*x* – 2202.059 (R = 0.99750) and *y* = 108741.195*x* – 2421.345 (R = 0.99770) for 1 and 2, respectively. An aliquot (1 μl) of each sample was injected into GC system and the FID areas of the peaks corresponding to 1 and 2 were measured. Contents of 1 and 2 in the examined extracts were calculated using linear regression equations and expressed as milligram per gram dry plant material (mg/g DM).

In this study, each experiment was performed in triplicate. All the data values were expressed as means with standard deviation.

## RESULTS AND DISCUSSION

UAE of alantolactone (1) and isoalantolactone (2) from *I. helenium* roots was studied. The effects of ethanol (EtOH) concentration, solid/solvent ratio, time, temperature and number of extraction steps on the extraction yields of both sesquiterpene lactones were investigated. Different extraction methods, including MSDE, maceration and infusion were also employed in order to compare the obtained results. A modified GC method[10] was used for simultaneous determination of the content of 1 and 2 in investigated extracts.

Chloroform has been successfully used as the solvent for extraction of sesquiterpene lactones, but because of its high toxicity in the present investigations, EtOH was adopted as the solvent.

**Figure 2:**
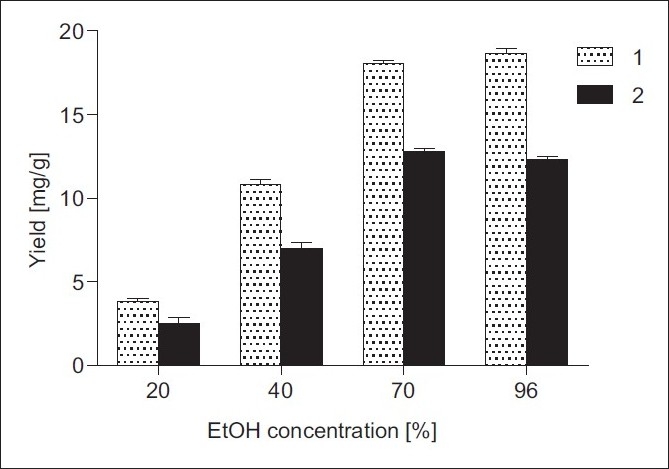
Effect of EtOH concentration on the yields of 1 and 2 from *I. helenium* roots: solid/solvent ratio 1:20 (w/v), temperature 25°C, UAE time 30 min

Initially, the effect of the EtOH concentration on the yields of 1 and 2 was studied, keeping the solid/solvent ratio 1:20 (w/v) in ultrasonic cleaning bath at 25°C. Figure 2 shows that increasing EtOH concentration results in higher extraction yields of 1 and 2. It could be explain with nonpolar nature of the investigated sesquiterpene lactones. Thus, the yields of 1 and 2 reached 18.04 and 12.77 mg/g in 70% EtOH from 3.81 and 2.47 mg/g in 20% EtOH. Slight raise in the yield of 1 (18.65 mg/g) and drop in the amount of 2 (12.31 mg/g) were observed when extraction was performed with 96% EtOH.

**Figure 3:**
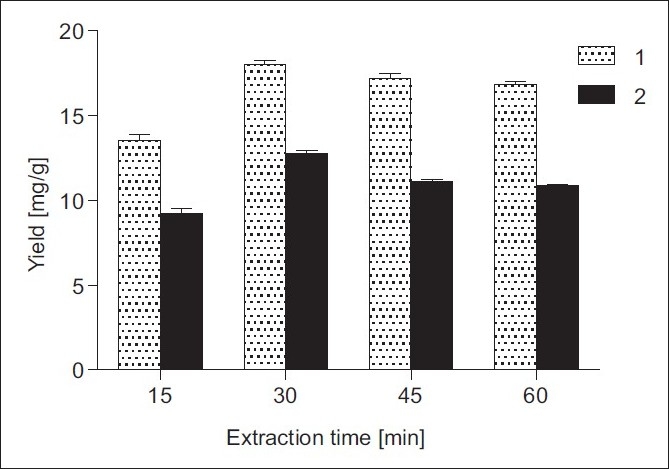
Effect of extraction time on the yields of 1 and 2 from *I. helenium* roots: 70% EtOH, solvent/solid ratio 1:20 (w/v), extraction temperature 25°C

Further, the effect of ultrasonic time on the yields of 1 and 2 was also studied. As can be seen from Figure 3, the amounts of 1 and 2 enhanced rapidly for the first 30 min of ultrasonic irradiation and then decreased slowly to 16.83 and 10.87 mg/g for 60 min. Consequently, the optimum extraction time for lactones 1 and 2 under ultrasonic conditions was found to be 30 min.

**Figure 4:**
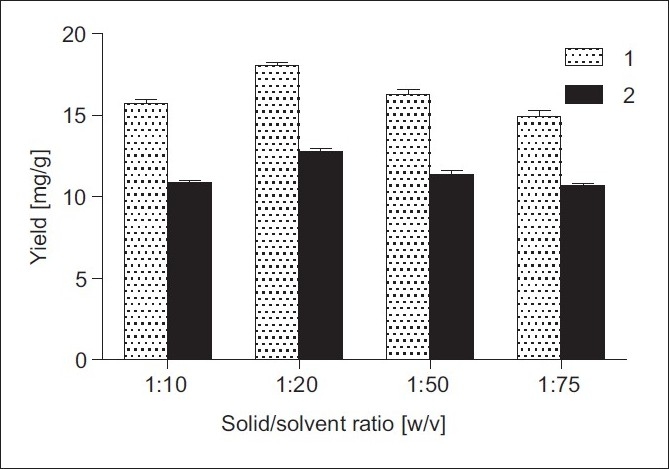
Effect of solid/solvent ratio (w/v) on the yields of 1 and 2 from *I. helenium* roots: 70% EtOH, extraction temperature 25°C, UAE time 30 min

The effect of different solid/solvent ratios [1:10, 1:20, 1:50 and 1:75 (w/v)] at working temperature 25°C was also examined. Figure 4 indicates that a larger solvent volume does not lead to higher sesquiterpene lactone yields. The obtained results demonstrated that a solid/solvent ratio of 1:20 (w/v) was the most beneficial condition for effective extraction of alantolactone and isoalantolactone from *I. helenium* roots.

**Figure 5:**
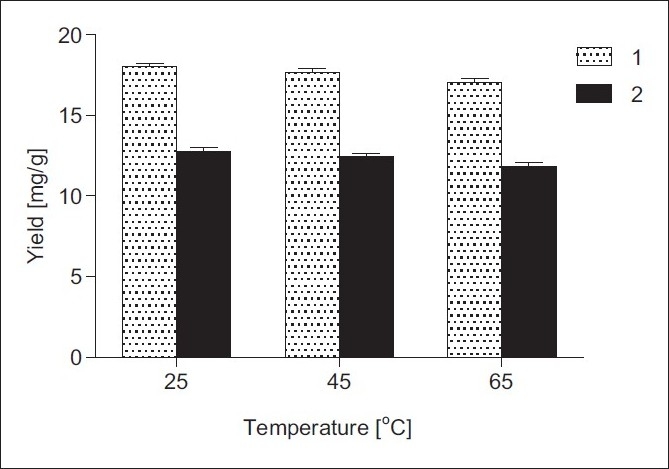
Effect of extraction temperature on the yields of 1 and 2 from *I. helenium* roots: 70% EtOH, solvent/solid ratio 1:20 (w/v), UAE time 30 min

The influence of the temperature on the extraction yields of 1 and 2 was also investigated and the results are summarized in Figure 5. As can be seen, the extraction temperature did not significantly affect the yields of 1 and 2. Moreover, a slight reduction in the yields of extracted sesquiterpene lactones at higher temperatures could be attributed to thermodegradation. Obviously, the optimum extraction temperature in this investigation was 25°C.

**Figure 6:**
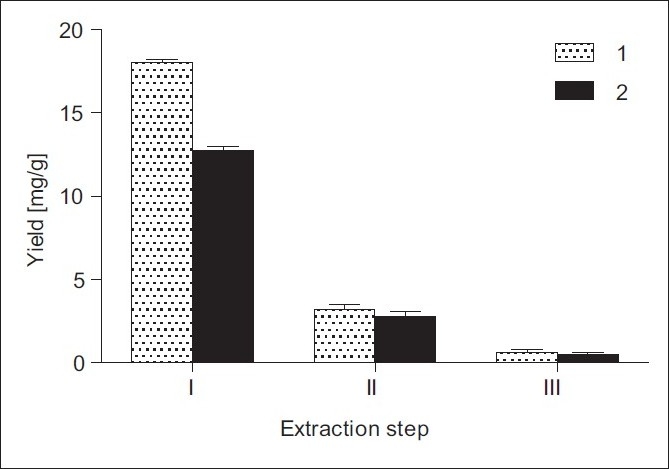
Effect of extraction steps on the yields of 1 and 2 from *I. helenium* roots: 70% EtOH, solvent/solid ratio 1:20 (w/v), extraction temperature 25°C, UAE time 30 min

In general, the extraction amount of the constituents increased with the increase in the extraction steps. In order to investigate the influence of number of extraction steps on the yields of 1 and 2, ground sample was extracted three times (3 × 30 min) under the above optimized conditions, i.e., in 70% EtOH, solid/solvent ratio 1:20 (w/v) for 30 min at 25°C [Figure 6]. It was found that the major amounts of 1 and 2 were extracted at the first extraction step, significantly lower amounts at the second step and only 0.6 and 0.5 mg/g of 1 and 2, respectively, at the third step.

**Figure 7:**
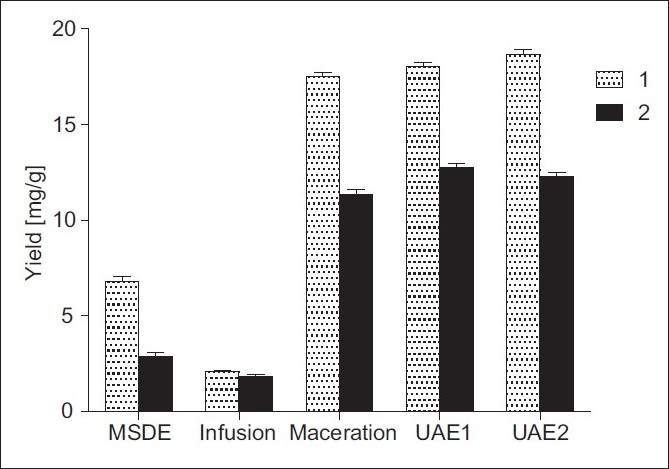
Comparison of different extraction methods on the yields of 1 and 2 from *I. helenium* roots: MSDE 4 hours; infusion – boiling H_2_O, 10 min; maceration – 70% EtOH, 24 hours, room temperature; UAE1 – ultrasound-assisted extraction in 70% EtOH, 30 min, 25°C; UAE2 – ultrasound-assisted extraction in 96% EtOH, 30 min, 25°C

Finally, the results of UAE were compared with those obtained by other classical methods – maceration at room temperature, MSDE and infusion. Thus, the amounts of 1 and 2 [Figure 7] in infusion tea and essential oil were significantly lower than those obtained by maceration. It is worthy to mention that the amounts of 1 and 2 under UAE were higher or almost equal to those obtained by classical maceration but the extraction time was significantly shorter.

## CONCLUSION

The results obtained show that UAE is an efficient method for extraction of alantolactone and isoalantolactone from *I. helenium* roots. UAE results in higher yields of sesquiterpene lactones, reduction in extraction time and decrease in temperature, which is particularly favorable for the extraction of thermally unstable compounds from plant materials.
